# Protocol for building and using a maximum power point output tracker for perovskite solar cells

**DOI:** 10.1016/j.xpro.2024.103394

**Published:** 2024-10-17

**Authors:** Arturo Sanz-Marco, Rodrigo Jeronimo-Cruz, Marta Haro, Emilio J. Juarez-Perez

**Affiliations:** 1 Instituto de Nanociencia y Materiales de Aragón (INMA), CSIC-Universidad de Zaragoza, 50009 Zaragoza, Spain; 2 Aragonese Foundation for Research and Development (ARAID) Government of Aragon, 50018 Zaragoza, Spain

**Keywords:** energy, material sciences, computer sciences

## Abstract

We recently developed a galvanostatic maximum power point output (MPPT) algorithm for high-hysteresis perovskite solar cells (PSCs), enabling continuous and precise power tracking. Here, we present a protocol for assembling the tracker, implementing the algorithm on a microcontroller, and conducting JV scans and stabilized output power (SOP) or traditional perturb and observe (P&O) tracking for small-area photovoltaic cells. We also describe steps for collecting, storing, and plotting data and explain the device’s operational modes and functions.

For complete details on the use and execution of this protocol, please refer to Juarez-Perez et al.^1^

## Highlights

• Steps to build a maximum power point tracker
• Guidance on the calibration process of the device
• Instructions for setting up the device in different operational modes
• Plotting data as produced or stored for further processing

## Abstract (editor highlights)

Publisher’s note: Undertaking any experimental protocol requires adherence to local institutional guidelines for laboratory safety and ethics.

## Graphical abstract

**Figure:**
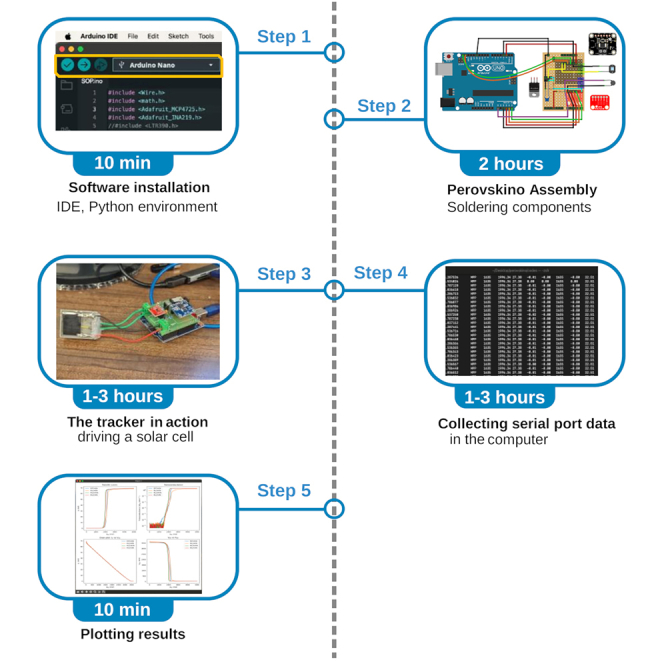

## Before you begin

**Figure 1:**
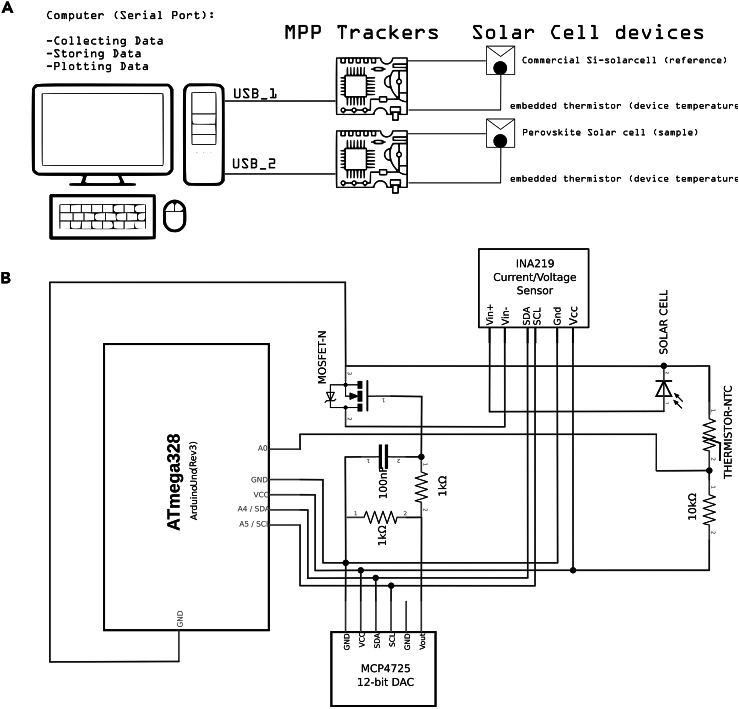
Solar tracking system components and electrical connections (A) General overview of the system usage consisting of PC, two trackers and two solar cells. (B) Arduino UNO and Perovskino shield (the tracker) device connection scheme.

Researchers investigating emerging solar cell technologies, such as perovskite solar cells (PSCs), often face challenges when conducting extended operational stability assessments due to the limited availability of cost-effective equipment. While determining solar cell efficiency through acquiring a JV curve can be swiftly accomplished within seconds or minutes using a basic potentiostat under controlled illumination, evaluating the operational stability of these devices, particularly by tracking the maximum power point output (MPPT), requires prolonged time-consuming use of costly potentiostat and solar simulator equipments. These equipments are often in high demand for other researching tasks, making it difficult to dedicate them solely to stability assessments. To address this challenge, we have developed a protocol that allows for a preliminary screening of the best performing solar cells without significant economic and space impact in the laboratory. This protocol involves the use of a simple tracker and their operational modes that enable the gathering of statistically significant stability data into a computer (Figure 1A). Our recent work introduced a galvanostatic MPPT algorithm that offers continuous and precise power tracking, enhancing the performance, particularly of challenging high-hysteresis PSCs, which is crucial for propelling PSCs closer to commercial feasibility.

## Key resources table

**Table:** 

REAGENT or RESOURCE	SOURCE	IDENTIFIER
**Software and algorithms**		

Arduino IDE	Arduino IDE 2.3.2 Windows/macOS/Linux	https://www.arduino.cc/en/software
GitHub repository	Perovskino: A Maximum Power Point Tracking (MPPT) algorithm	https://github.com/ej-jp/perovskino/releases/tag/v0.1
Miniconda	Miniconda Windows/macOS/Linux	https://docs.anaconda.com/free/miniconda/index.html

**Other**		

8-bit MC (ATmega328) as found in Arduino UNO (Rev3) or equivalent	Arduino	https://www.arduino.cc/en/hardware
12-bit digital-to-analog converter breakout (MCP4725)	Digikey	https://www.digikey.es/es
N-channel MOSFET (IRLZ34N)	Infineon Technologies	https://www.mouser.es/manufacturer/infineon/
12-bit digital power monitor breakout (INA219)	DigiKey	https://www.digikey.es/es
NTC 10 kOhm thermistor (MF52)	DigiKey	https://www.digikey.es/es
100 nF ceramic capacitor	DigiKey	https://www.digikey.es/es
2 × 1 kOhm resistors	DigiKey	https://www.digikey.es/es
10 kOhm resistor	DigiKey	https://www.digikey.es/es
Two screw terminal with 2 pins	DigiKey	https://www.digikey.es/es

## Step-by-step method details

### Software installation

**Timing: 10 min**

Here, we describe the steps for downloading and installing the software and algorithms needed before assembling the Perovskino.

1. Install Arduino IDE software and libraries for the breakouts.
  a. Go to https://www.arduino.cc/en/software and download the proper version according to the operating system of the computer (Windows/macOS/Linux).
  b. Once the Arduino IDE is installed, open the Library Manager tab and install the software libraries listed below:
    i. Install library “Adafruit MCP4725”.
    ii. Install library “Adafruit INA219”.
2. Download the algorithm code of the Perovskino.
  ***Note:*** The Perovskino firmware version used in this protocol and the previous publication was frozen as a first-release version and deposited in the Zenodo repository.
  a. Download and unzip the file in https://doi.org/10.5281/zenodo.10647187 where you can find all the scripts and files mentioned in this protocol. This release also can be reached in the GitHub repository under this link https://github.com/ej-jp/perovskino/releases/tag/v0.1.
    ***Note:*** This firmware is a work in progress. Well tested improvements will be released as new versions or releases in the GitHub perovskino web page. The user also can test and/or contribute in this main repository but this is not recommended for deployment. If you still prefer to use this more updated repository, please follow the step 2.b below.
  b. Go to https://github.com/ej-jp/perovskino and download all the fold by ZIP. Or cloning the GitHub repository, by executing the following command in your terminal (if you have installed Git in your computer).
    > git clonehttps://github.com/ej-jp/perovskino.
3. Install Miniconda and libraries.
  ***Note:*** For data acquisition and recording of serial data for further processing, an environment with a modern Python 3 is necessary. We use a minimal subset of Conda called Miniconda to manage this Python environment in the computer.
  a. Go to https://docs.anaconda.com/free/miniconda/index.html and download the proper version for your computer system (Windows/macOS/Linux).
  b. Install Miniconda on your computer following the steps recommended in the web page.
  c. Open the terminal and execute the next command to know if the installation was correct.
    > conda info
    ***Note:*** For Microsoft system, open the Anaconda Powershell system (miniconda3) as a Terminal.
    ***Note:*** For MacOS/Linux, open the default terminal system.
    ***Note:*** If the installation was correct, you will get all the information about your Miniconda version.
  d. Create a specific environment for the dependencies instead of the *base* environment called *perovskino-env.*
    > conda create -n perovskino-env python=3.12.2 pyserial=3.5 pandas=2.2 matplotlib=3.8.4 scipy=1.12.0
  e. Check that the new environment was created with the next command.
    ***Note:*** If the new environment was correctly created, this command shows “perovskino-env” and its binaries route.
    > conda env list
  f. Active the new environment with the next command.
    ***Note:*** The new environment is activated if the line command of the terminal starts with the string (perovskino-env) user@computer:∼$
    > conda activate perovskino-env

### Assembling the perovskino shield for the arduino UNO device

**Timing: 2 h**

This section provides a guide for assembling the Perovskino shield. An Arduino UNO device and this shield coupled on top constitute the MPP tracker.

4. Assemble the tracker device connections as shown in schematic of Figure 1B.

**Figure 2:**
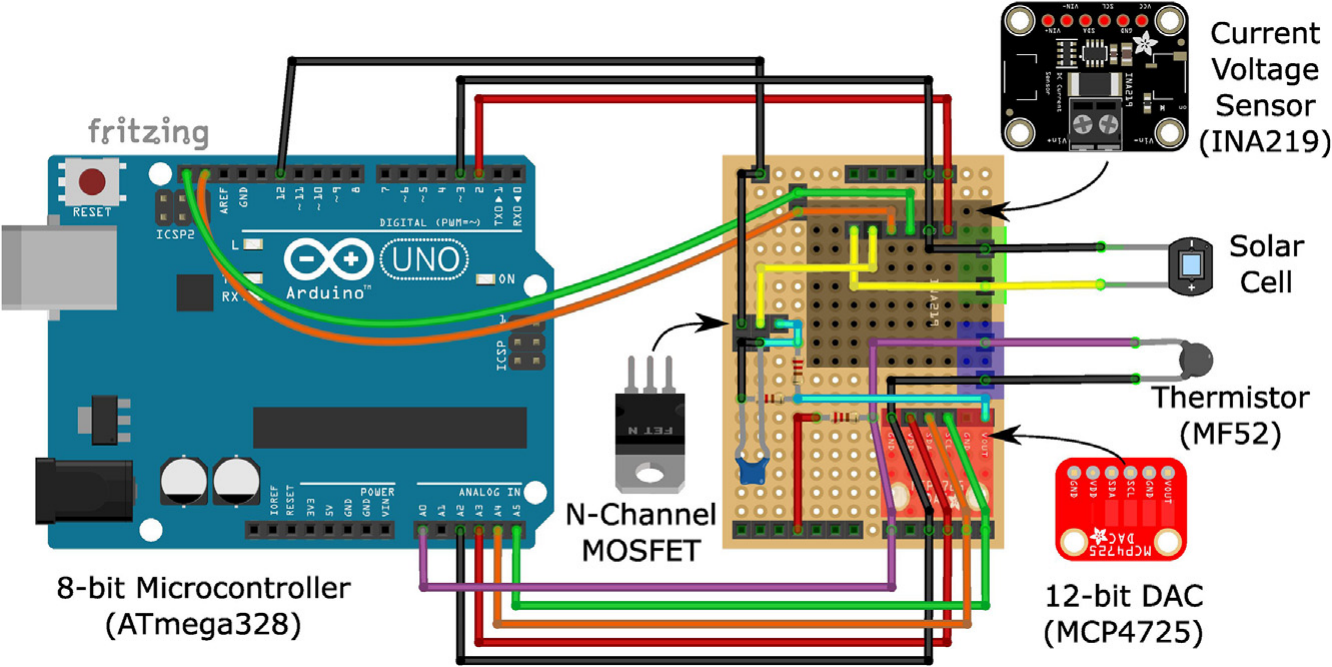
Perfboard version assembling scheme of the Perovskino shield

***Note:*** An example of Perovskino shield assembled in perfboard is shown in Figure 2.

***Note:*** The perfboard version of the Perovskino used digital and analog outputs to power the INA219 and MCP4725 breakouts. A newer and more convenient version of the Perovskino shield was specifically designed in PCB board and it is available at Aisler https://aisler.net/p/SDRQAOGS under demand. Also, the Gerber files can be found in the Zenodo or GitHub 0.1 Perovskino firmware release. Finally, a full assembled tracker (Perovskino shield + Arduino UNO) or only the PCB edition of the Perovskino shield with breakouts and components as employed in this study is available on request, but we may require a payment and/or a completed materials transfer agreement if there is potential for commercial application.

**Figure 3:**
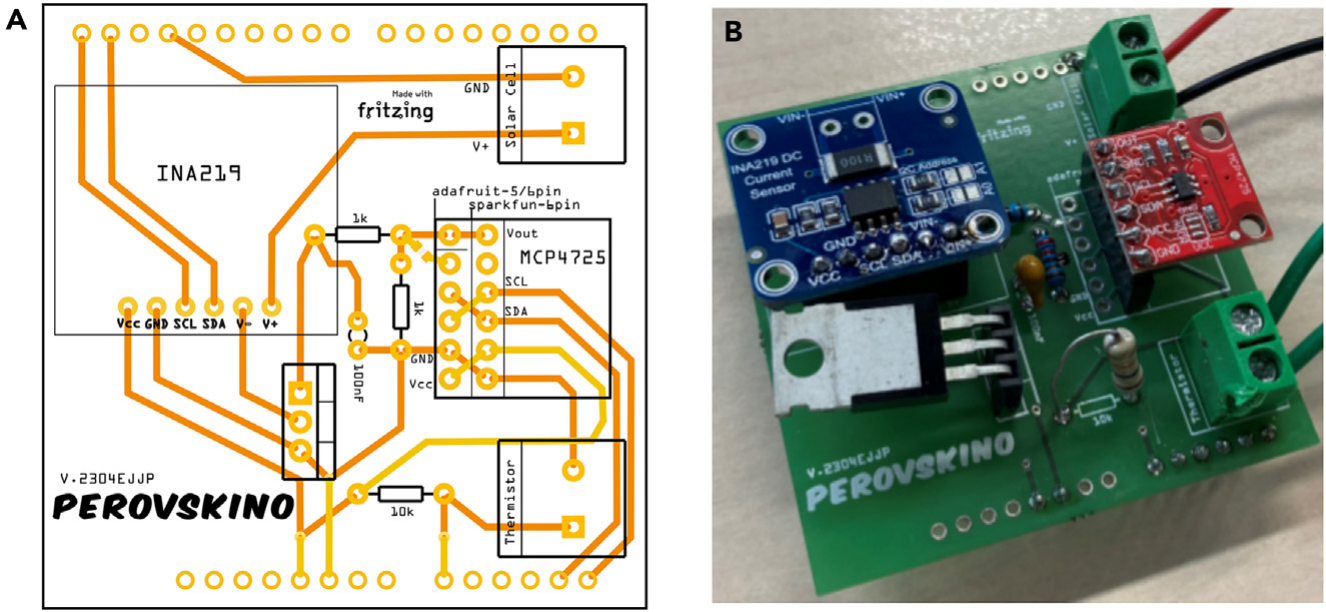
The PCB version of the Perovskino shield (A) Perovskino shield PCB board scheme and (B) picture of the shield with assembled components.

***Note:*** The PCB version board is dimensioned so it can be connected directly on the top of the Arduino device. The location of each component is clearly specified in the PCB board. The PCB board scheme and a picture of the components assembled in it are shown in Figure 3.

***Note:*** This PCB board can accommodate various commercially available versions of the MCP4725 breakout. If employing the 5-pin Adafruit pin configuration, solder the pad connecting VGATE and pin 5.

**Figure 4:**
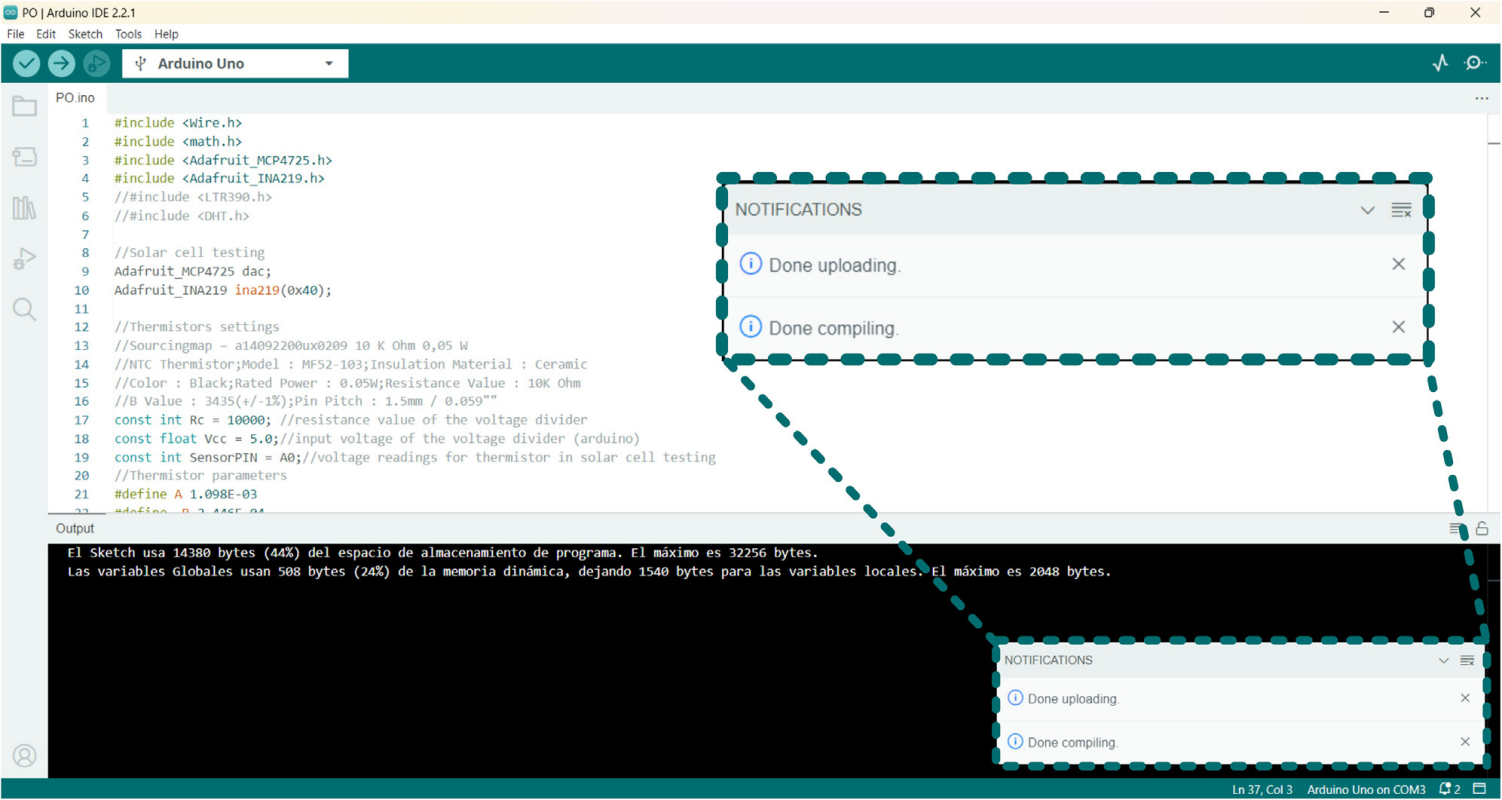
Screenshot of the Arduino IDE window After compiling and loading the algorithm to the Arduino, a notification will pop-up (zoomed in the inset for an easier reading).

**Figure 5:**
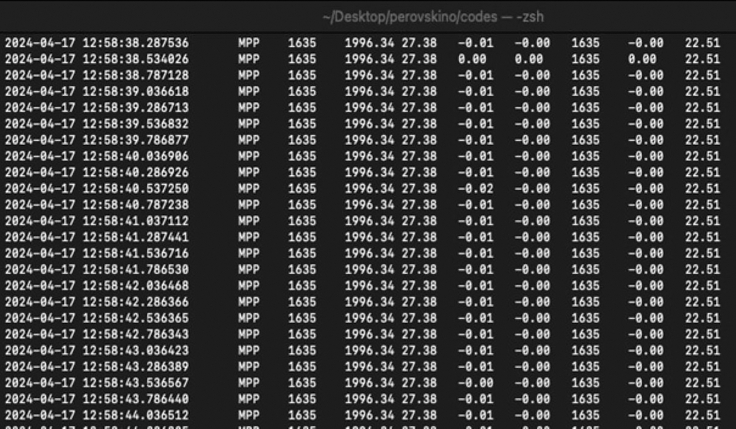
Screenshot of the Terminal window showing the data collection while running the *“capture-datos-mpp-temperature.py”* script

### Upload the firmware to the arduino microcontroller

**Timing: 10 min**

Here, we describe the steps needed to upload firmware to the microcontroller.

***Note:*** After assembling the Perovskite shield and connecting it to the Arduino board, the initial recommended step would involve uploading an I2C address checker using the Arduino IDE to scan the I2C bus. This scan aims to identify the addresses assigned to the INA219 and MCP4725 breakouts on the Perovskino shield. It is essential to modify if necessary these addresses in the .ino files. For instance, the addresses for our breakouts were 0 × 40 and 0 × 60 for the INA219 and MCP4725, respectively. A useful tool for finding I2C device addresses can be found here https://playground.arduino.cc/Main/I2cScanner/.

5. Download the i2c_scanner.ino file from this URL (https://playground.arduino.cc/Main/sourceblock_1/index.txt?action=sourceblock&num=1).
6. Open the i2c_scanner.ino file using the Arduino IDE software.
7. Connect the power tracker (Perovskino shield + Arduino UNO) to the computer with a USB cable.
8. Select the Arduino UNO board and the appropriate COM/Serial Port from the top menu of the Arduino IDE.
9. Validate the code for any errors (e.g., absence of libraries) by compiling it using the button located in the top-left corner. Upload the code with the upload button in the top-left corner (Figure 4).
10. Open serial monitor to check that the power tracker release data to the serial data port.
11. Record the I2C addresses assigned to the INA219 and MCP4725 breakouts.

***Note:*** To identify the COM/Serial port without doubts, disconnect the Arduino and then reconnect it to observe the appearance of the new COM/Serial port.

***Note:*** Once the breakout I2C addresses have been identified and the .ino files variables storing these values were modified if their I2C addresses were different, any of the five operational modes firmware offered in the initial release of Perovskite v0.1 can be employed. However, it is recommended to upload the INA219 voltage and current calibration procedure to the microcontroller when using the tracker for the first time.

### General overview of the python scripts in the PC side collecting, storing and plotting data from the tracker

**Timing: 1–3 h**

In this section, we describe the steps for the general process for obtaining, storing and plotting the data obtained by the Perovskino.

***Note:*** The Serial Monitor window in the Arduino IDE is the first step to check that the firmware was correctly uploaded in the MC. Data displayed in this window could already be used with simple copy and paste operations into our preferred program for plotting. However, we wanted to accompany the tracker with a series of Python scripts that automate all these tasks.

***Note:*** As a general rule, all scripts starting with the suffix “capture-...” are scripts that collect data lines from the serial port and save them in CSV files in the *dataraw* folder by default. Scripts of this type run in parallel while the tracker is collecting data.

***Note:*** A common step before running these Python scripts is the activation of the proper environment. Open the terminal and activate the new environment previously created with the next command.

> conda active perovskino-env

***Note:*** For Microsoft system, open the Anaconda Powershel system (miniconda3) as a Terminal.

***Note:*** For MacOS/Linux, open the default terminal system.

12. Collecting and storing data.
  a. Identify the COM/Serial port using the tracker device plugged into the computer. It will depend on which operating system you use.
    i. For Windows/macOS/Linux, identify the COM/Serial port that appeared previously on the Arduino IDE software. In case of macOS/Linux system changing the prefix “cu.∗” instead “tty.∗”. In case of windows will appear as a “COM#”.
    ii. For macOS/Linux in the terminal, execute the next command.
      > ls /dev/tty.∗
  b. For Windows in the terminal, execute the next command.
    > chgport
  c. Edit the python script *“capture-datos-mpp-temperature.py”* in the directory perovskino-0.1/codes/02_MPP-algorithms/capture_and_grapher with the correct “portpc” (line 7–9).
    ***Note:*** For Windows system the name of the portpc is as a “COM#”. For macOS/Linux system the name of the portpc is as a “/dev/tty.∗”.
    ***Note:*** In the script code “capture…” other portpc appear by default, for disability these lines code you can comment starting the line with “#” symbol.
    ***Note:*** To edit the python script, you can do it directly in the terminal using text editor as “vim” for macOS/Linux system or “notepad” editor for Windows or opening directly the file into the directory.
  d. Execute the python script *“capture-datos-mpp-temperature.py”* in the directory with the next command on the terminal.
    > python capture-datos-mpp-temperature.py
    ***Note:*** All captured data is saved in a file .csv in the directory “perovskino-0.1/codes/02_MPP-algorithms/capture_and_grapher/dataraw”
    ***Note:*** The starting of the data collection will be observed with the constant refresh of the screen with data in 9 columns, as shown in Figure 5.
  e. To stop the script use de command ctrl/control + C.
13. Plotting data.
  ***Note:*** In addition to the "capture" type scripts, we developed the "grapher-" type scripts to plot the data collected in these CSV files. These scripts are run once the serial port data collection is finished, with the exception of the “Alive” “grapher-“ type scripts which are able to plot data as it is collected and thus have a closer look at the performance of the solar tracker-cell pair.
  a. Plotting data during data collection using a python script.
    i. In another terminal window, active the new environment previously created with the next command.
      > conda active perovskino-env
    ii. Execute the python script perovskino-0.1/codes/02_MPP-algorithms/capture_and_grapher/grapher4_alive.py.
      > python grapher4_alive.py
    iii. To stop the script use de command ctrl/control + C.
  b. Plotting data after finishing the data collection using a python script.
    i. In another terminal window, activate the new environment previously created with the next command.
      > conda active perovskino
    ii. execute the python script perovskino-0.1/codes/02_MPP-algorithms/capture_and_grapher/grapher2.py.
      > python grapher2.py
    iii. To stop the script use de command ctrl/control + C.
  c. Use your preferred program to plot and process the collected data saved in a file .csv in the directory “perovskino-0.1/codes/02_MPP-algorithms/capture_and_grapher/dataraw”

### Perovskino checks prior to use

**Timing: 1–3 h**

In this section, we describe the steps to follow to assure your Perovskino is working properly.

**Figure 6:**
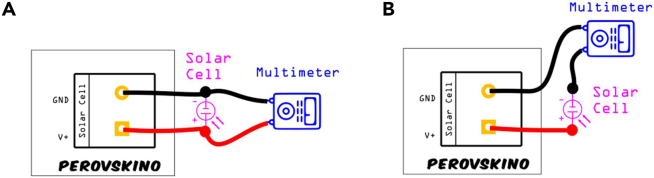
Connection wiring scheme for the INA219 calibration procedure Connection wiring for (A) voltage and (B) current calibration using a solar cell and the multimeter.

14. INA219 voltage and current calibration.
  ***Note:*** The calibration procedure needs a reliable multimeter between the solar cell and INA219 in the shield.
  a. Assemble the complete tracker (Perovskino shield + Arduino UNO) and connect a silicon solar cell or other stable solar cell.
  b. Place the multimeter correctly in relation to the solar cell for calibration measurements, either in parallel for voltage or in series for current calibration, as shown in Figure 6.
  c. Upload the file "INA219-calibration-ino.ino" in the Perovskino editing this file depending on the type of calibration (voltage or current calibration). In any case, confirms that calibration parameters *correcV* and *correcI* parameters are 0 and 1, respectively.
  d. For voltage calibration, configure the MCP4725 to a voltage below the threshold voltage of the MOSFET to achieve an open circuit condition (i.e., *countermpp = 1*) in the solar cell and place the multimeter as indicated in Figure 6A.
  e. Open the Serial Monitor in the Arduino IDE and vary light irradiation (5–6 different irradiation powers to obtain 5–6 different points for the calibration) while noting the voltage values from both the multimeter screen and serial monitor window.
  f. Determine the correct voltage parameter (*correcV*) as the ordinate at the origin from linearly fitting the multimeter and INA219 data, using a line equation with a slope of 1.
  g. For current calibration, set the MCP4725 to a voltage higher than the threshold voltage of the MOSFET (i.e., *countermpp = 4095*) to induce a short circuit condition in the solar cell and place the multimeter as indicated in Figure 6B.
  h. Open the Serial Monitor in the Arduino IDE and vary light irradiation (5–6 different irradiation powers to obtain 5–6 different points for the calibration) while noting the current values from both the multimeter screen and Serial monitor window.
  i. Calculate the *correcI* as the slope derived from linearly fitting the multimeter and INA219 data.
15. MOSFET Checker.
  a. Assemble the complete tracker (Perovskino shield + Arduino UNO) and connect a silicon solar cell or other stable solar cell under light irradiation close to 1 SUN or 100 mW/cm^2^.
  b. Utilize the Arduino IDE to upload the file mosfet-checker-ino/mosfet-checker-ino.ino.
  c. Connect the MOSFET for evaluation.
  d. Start data acquisition by running “capture_and_grapher/capture-datos.py”. Edit the script to reflect the correct serial USB port on the computer assigned to the tracker device, if needed.
  e. Generate the graphs based on the collected data running “capture_and_grapher/grapher2.py”.

## Expected outcomes

### Perovskino operational modes

This section outlines the main operational modes of the Perovskino shield and the expected obtained results for each operational mode. After installing the necessary software in the computer and assembling the power tracker (Perovskino shield + Arduino UNO), the next tasks include testing its basic functionality, and confirming the correct operation of all components. This procedure may involve connecting a single solar cell to the tracker under a light source for testing purposes. The first step, common to all five operational modes of the shield, involves uploading the firmware associated with the intended operational mode to the Arduino microcontroller.

### INA219 voltage and current calibration

**Figure 7:**
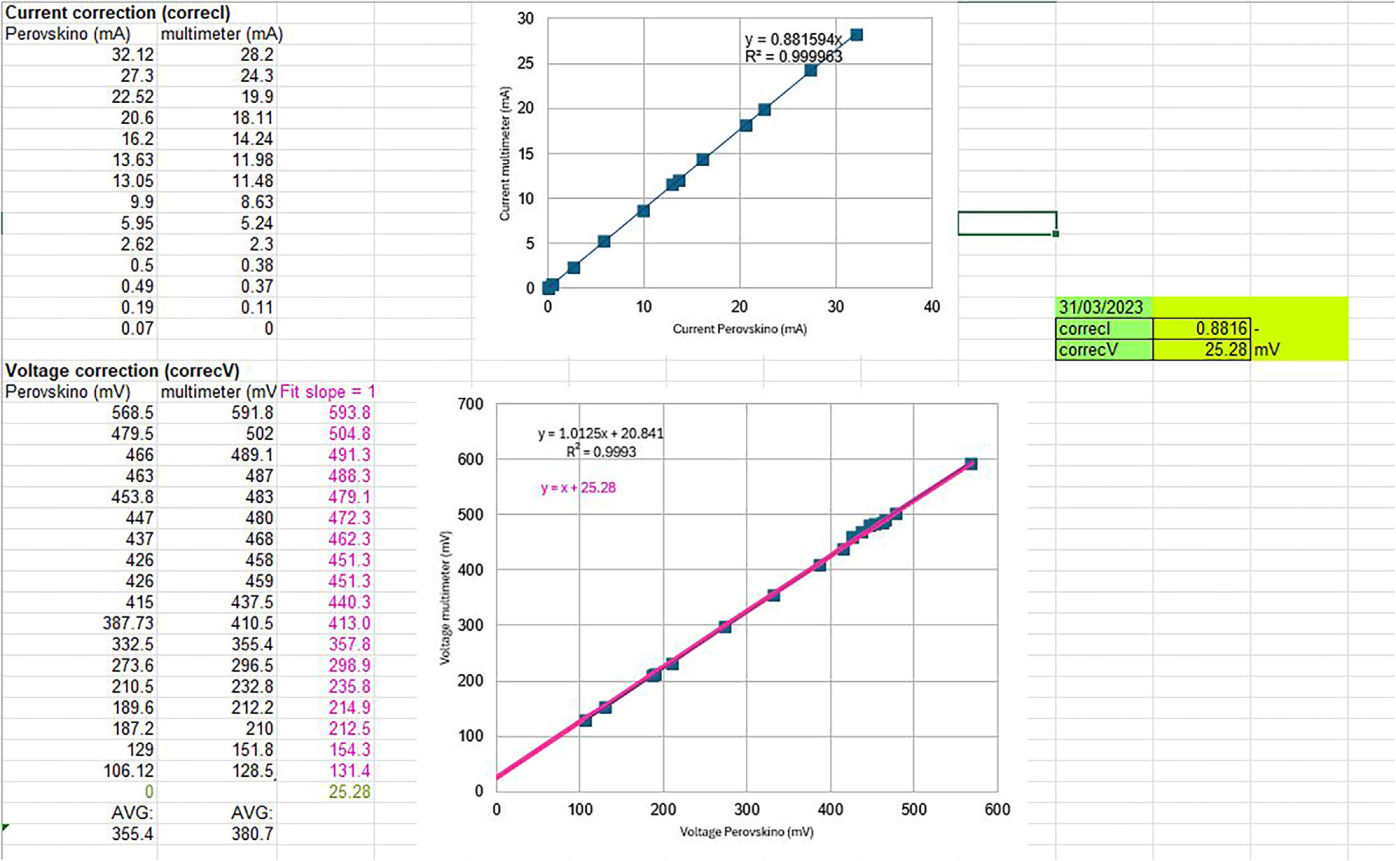
Current correction and Voltage correction fitting process example and *correcI* and *correcV* factors values obtained using the “INA219_calibration.odes” template file included in the downloaded folder in Step 3

As described in step 13 in step-by-step section, you will obtain a voltage correction factor (*correcV*) as the ordinate at the origin from linearly fitting the multimeter and INA219 data, using a line equation with a slope of 1; and a current correction factor (*currentI*) as the slope derived from linearly fitting the multimeter and INA219 data. Refer to the "INA219_calibration.ods" file for an example, as shown in Figure 7.

***Note:*** Instead of capturing data directly from the Serial Monitor window in the Arduino IDE, a data acquisition by executing the "capture-datos-INA219_calibration.py" script recording the data in csv files can be carried out. Data visualization can be performed using a "grapher" python script in the calibration folder.

### MOSFET checker

**Figure 8:**
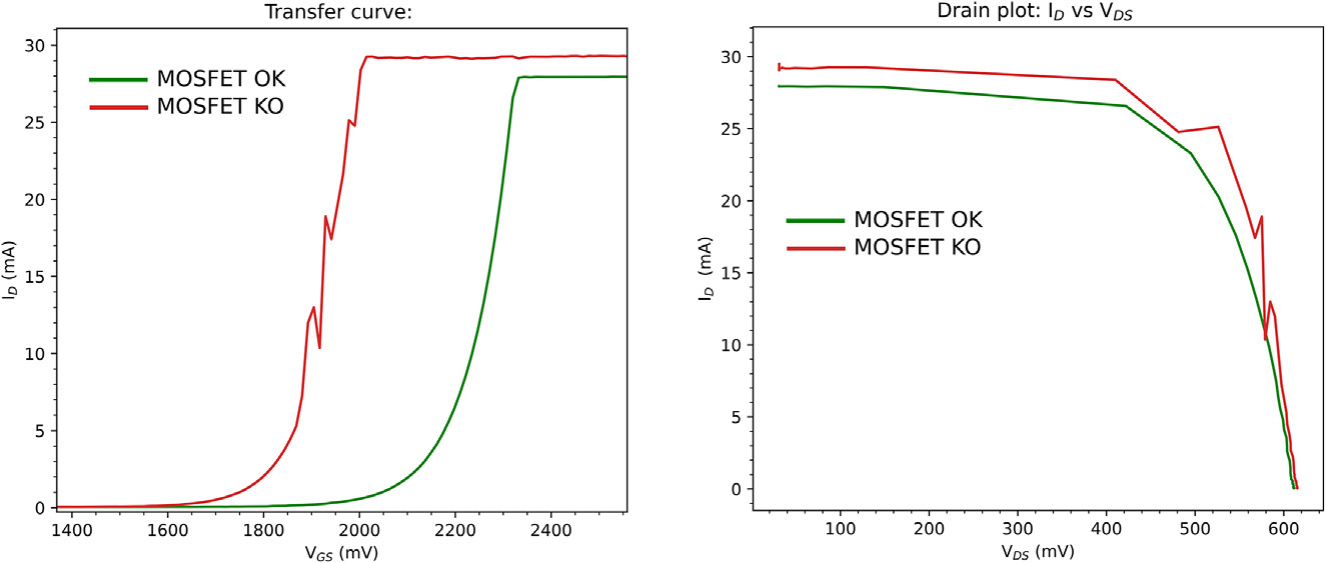
Example of bad (red) and good (green) condition MOSFET for the Perovskino Shield

To illustrate an example of a broken or not OK MOSFET and a good one, please check Figure 8 the transfer curve and JV curve traced using an example of both.

***Note:*** For the INA219 calibration of the Perovskino shield, the N-MOSFET was set to induce open or short circuit conditions in the solar cell by adjusting the integer *countermpp* variable to 1 or 4095 in the MCP4725 DAC, which controls the gate voltage of the MOSFET. However, verifying the proper function of the MOSFET in the range of interest between these two extreme values is necessary for generating smooth JV curves and efficiently driving the MPPT routines. The MOSFET-Checker operational mode ensures that the selected N-Channel MOSFET does not exhibit glitches or noise when the gate voltage is controlled by the MCP4725 DAC. This check should be performed before running the tracker device and for periodic maintenance.

### JV-SOP operational mode

Upon the calibration and MOSFET checker routines validates the correct functioning of the tracker, it is time to run a basic experiment tracking the maximum power output of well-behave (no JV hysteresis) solar cell like a silicon solar cell. This basic MPPT algorithm alternates a JV measurement stage to determine the voltage of the cell delivering the maximum power (V_MPP_) and then sets the solar cell voltage at this V_MPP_ value during a determined time interval (SOP: Stabilized Output Power) stage. This basic MPPT routine repeats the JV and SOP steps cyclically and infinitely.

As before during calibration or MOSFET checking operational modes, the first step to run this routine is to upload the firmware in the .ino file in the folder perovskino-0.1/codes/02_MPP-algorithms/SOP to the tracker. Modify the cycling time of each stage as needed in this .ino file.

As an example of this operational mode, refer to Figure 3 in ref.,^1^ which validates the galvanostatic approach in a Si-cell. The figure displays the JV, power vs. cell voltage, and MOSFET gate voltage vs. cell voltage curves obtained with a Si solar cell. The maximum power released by the cell is obtained in a specific gate voltage in the MOSFET which set indirectly the V_MPP_ for the cell. This gate voltage is maintained during the subsequent SOP stage.

***Note:*** There is another algorithm in the Perovskino perovskino-0.1 code release called SOP-Manual where the V_GATE_ can be modified on-the-fly manually on demand to observe the effect in the power delivered by the solar cell or try to find the V_MPP_ manually.

This naive JV-SOP MPPT routine is limited to research contexts for short-term (minutes or hours) stability tests using constant irradiation where JV derivable PV parameters such as V_OC_, J_SC_, FF, R_SERIES_ and R_SHUNT_ need of specific monitoring over time. Practical MPPT algorithms in real-world applications do not require a full JV curve analysis for MPP determination, as this approach is time-consuming and results in suboptimal power production from the solar cell. Also, the search for the MPP in a perovskite-based solar cell with high hysteresis needs a forward and a backward voltage scan JV curve before the SOP stage. In the next section, the P&O MPPT routine and its special application for high hysteresis perovskite solar cell will be discussed.

### P&O operational mode

In a perovskite solar cell, the hysteresis phenomenon produces a significant difference in the V_MPP_ determined from JV curves if the voltage sweep has been done forward (FWD, from J_sc_ to V_oc_) or backward (BWD, from V_oc_ to J_sc_), refer to Figure 4 in ref. ^1^ as an example of this behavior. This, in principle, unpredictable behavior makes it nearly impossible or results in high uncertainty when setting the solar cell at its maximum power point. However, the galvanostatic approach implemented in the firmware and running in this tracker overcomes this problem with the strategy explained in our recent article. Briefly, the voltage applied to the cell is controlled by the N-MOSFET, working as a variable resistor. The optimal V_GATE_ setting V_MPP_ in the solar cells is the value at which the maximum difference between the BWD and FWD appears, but setting the value to the average of the V_GATE_ for each FWD and BWD maximum power points leads to a more robust and fast method for both non-hysteresis and high hysteresis devices.

The MPP determination of the P&O routine consists of two steps: firstly, a BWD and FWD JV scan is made to determine both V_GATE_ producing the maximum output power for each scan. Then, the current established as a setpoint for the galvanostatic control of the MPP tracking will be determined as the average of these V_GATE_. After obtaining this value, the P&O operational algorithm starts for 3 h. The P&O stage modulates the V_GATE_ applied in order to maintain the maximum power output. As an example of this operational mode refer to Figure 10 in ref. ^1^ where this algorithm controls exceptionally well a high hysteresis perovskite solar cell under a EN-50530-type variable illumination conditions.

The .ino file of this operational mode can be modified to use other cycling stage times and other parameters setting the P&O algorithm as needed. Finally, other algorithms for the track of the maximum power delivered by the solar cell can be implemented by the user.

## Limitations

The parameters limiting the operational range of the Perovskino shield are established by the digital power monitor (INA219) component. This component cans measure up to **+26V** of solar cell voltage and up to **±3.2A** current measurement. The relevant line in the .ino file setting the resolution of this INA219 is the *void setup(){* section. By default, we use *ina219.setCalibration_16V_400mA()* setting in small single devices.

For measuring solar cells (modules and panels) that work at higher voltages and/or currents there are two possibilities:Substitute the INA219 component for another that can measure higher voltages, like the Adafruit **INA228** that can measure voltages up to **+85V** and currents up to **10A**or connect the solar cell to the INA219 through a voltage divider.

For the first option, a minimal change in the PCB is required because INA219 and INA228 have a different order and number of pins in their respective breakouts. For the second option, we have available a scheme for a breakout to install on the top of the Perovskino shield using the INA219. We have been able to track PSC modules releasing 39 V of V_OC_ and Copper Indium Gallium Selenide (CIGS) module releasing >3.2 A of I_sc_ under full real Sun using this second approach.

## Troubleshooting

### Problem 1

In Step 7 an Arduino error shows: avrdude: ser_open(): can’t set com-state for "\\.\∗”.

### Potential solution

Reset the Arduino board holding down the white button for 10 s. Unplug and plug the USB cable.

### Problem 2

In Step 7 an Arduino error shows: serial.serialutil.SerialException: Cannot configure port, something went wrong.

### Potential solution

Verify and install an older o newer version of the usb serial driver.

### Problem 3

In Step 7 an Arduino error shows: Adafruit XXXXXXXXXX doesn’t found.

### Potential solution

Install missed libraries following Step 1.b.

### Problem 4

In Step 12.d a terminal error shows after executing “capture-datos-...py” type scripts: UnicodeDecodeError: ‘utf-8′ codec can’t decode byte …

### Potential solution

Clear the terminal with command “clear” and then execute again the *capture* Python script.

### Problem 5

In Step 12.d a Miniconda error shows: "ModuleNotFoundError: No module named ‘serial'" and is not solved after executing “pip install serial”.

### Potential solution

execute “pip install pyserial”.

## Resource availability

### Lead contact

Further information and requests for resources should be directed to and will be fulfilled by the lead contact, Emilio J. Juarez-Perez (ejjuarezperez@unizar.es).

### Technical contact

Technical questions on executing this protocol and general algorithm implementation should be directed to and will be answered by the technical contact, Emilio J. Juarez-Perez (ejjuarezperez@unizar.es).

### Materials availability

A full tracker or only the Perovskino shield used in this study will be made available on request, but we may require a payment and/or a completed materials transfer agreement if there is potential for commercial application.

### Data and code availability

The example data and code generated during this study are available at GitHub: https://github.com/ej-jp/perovskino/releases/tag/v0.1 The version of record is archived at Zenodo. DOI at Zenodo: https://doi.org/10.5281/zenodo.10647187.

## Acknowledgments

The authors acknowledge the funding support from MCIN/AEI/10.13039/501100011033 and European Union NextGenerationEU/PRTR for project grants PID2022-140516OB-I00 (E.J.J.-P. and M.H.), PID2019-107893RB-I00 (E.J.J.-P.), EIN2020-112315 (E.J.J.-P.), and PID2019-108247RA-I00 (M.H.); a Ramón y Cajal fellowship (RYC-2018-025222-I; M.H.); and CPP2022-009766 (E.J.J.-P., A.S.-M., and R.J.-C.). Also, the authors acknowledge the funding support from the Aragon Regional Government for the Program for Research Groups under grants T57_23R (E.J.J.-P., A.S.-M., and R.J.-C.) and E31_20R (M.H.) and CIBER-BBN, ICTS “NANBIOSIS” (E.J.J.-P., A.S.-M., and R.J.-C.).

## Author contributions

Conceptualization, E.J.J.-P.; software, E.J.J.-P.; writing – original draft, R.J.-C. and A.S.-M.; writing – review and editing, E.J.J.-P. and M.H.; supervision, E.J.J.-P.; funding acquisition, M.H. and E.J.J.-P.

## Declaration of interests

The authors declare no competing interests.
